# Gaussian process forecasts *Pseudogymnoascus destructans* will cover coterminous United States by 2030

**DOI:** 10.1002/ece3.9547

**Published:** 2022-11-27

**Authors:** Ashton M. Wiens, Wayne E. Thogmartin

**Affiliations:** ^1^ U.S. Geological Survey, Upper Midwest Environmental Sciences Center La Crosse Wisconsin USA

**Keywords:** cave‐hibernating bats, disease spread, epidemiology, Gaussian process, *Pseudogymnoascus destructans*, White‐nose syndrome

## Abstract

White‐nose syndrome has been decimating populations of several bat species since its first occurrence in the Northeastern United States in the winter 2006–2007. The spread of the disease has been monitored across the continent through the collaboration of many organizations. Inferring the rate of spread of the disease and predicting its arrival at new locations is critical when assessing the current and predicting the future status and trends of bat species. We developed a model of disease spread that simultaneously achieves high‐predictive performance, computational efficiency, and interpretability. We modeled white‐nose syndrome spread using Gaussian process variations to infer the spread rate of the disease front, identify areas of anomalous time of arrival, and provide future forecasts of the expected time of arrival throughout North America. Cross‐validation of model predictive performance identified a stationary Gaussian process without an additional residual error process as the best‐supported model. Results indicated that white‐nose syndrome is likely to spread throughout the entire continental United States by 2030. These annually updatable model predictions will be useful in determining the horizon over which disease management actions must take place as well as in status and trend assessments of disease‐affected bats.

## Graphical abstract

White‐nose syndrome has been decimating populations of several bat species since its first occurrence in the Northeastern United States in winter 2006‐2007. We developed a model of disease spread that simultaneously achieves high predictive performance, computational efficiency, and interpretability, allowing inference of the spread rate of the disease front, identification of areas of anomalous time of arrival, and providing future forecasts of the expected time of arrival throughout North America. Results indicated white‐nose syndrome is likely to spread throughout the entire continental United States by 2030, and annually updatable model predictions will be useful in determining the horizon over which disease management actions must take place as well as in status and trend assessments of disease‐affected bats.

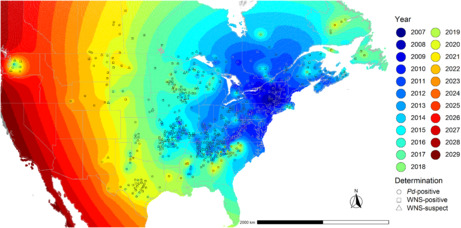

## 1 INTRODUCTION

Emerging infectious diseases (EIDs) in wildlife have been on the rise over the past few decades and contribute to the current biodiversity crisis (Daszak et al., 2000). EIDs also place conservation efforts in an acute state of crisis, often resulting in responses that are “too little, too late” (Voyles et al., 2015). Data‐supported conservation tools that are accessible, shareable, reproducible, and fast could help managers rapidly respond to EIDs.

Modeling and predicting the spatial spread of pathogens can improve understanding of patterns and drivers of disease across species, space, and time and for planning and prioritizing disease management and intervention strategies. However, spatial models can be complex and computationally intensive, making it difficult to reproduce, to update over time, and to share among practitioners.

**FIGURE 1:**
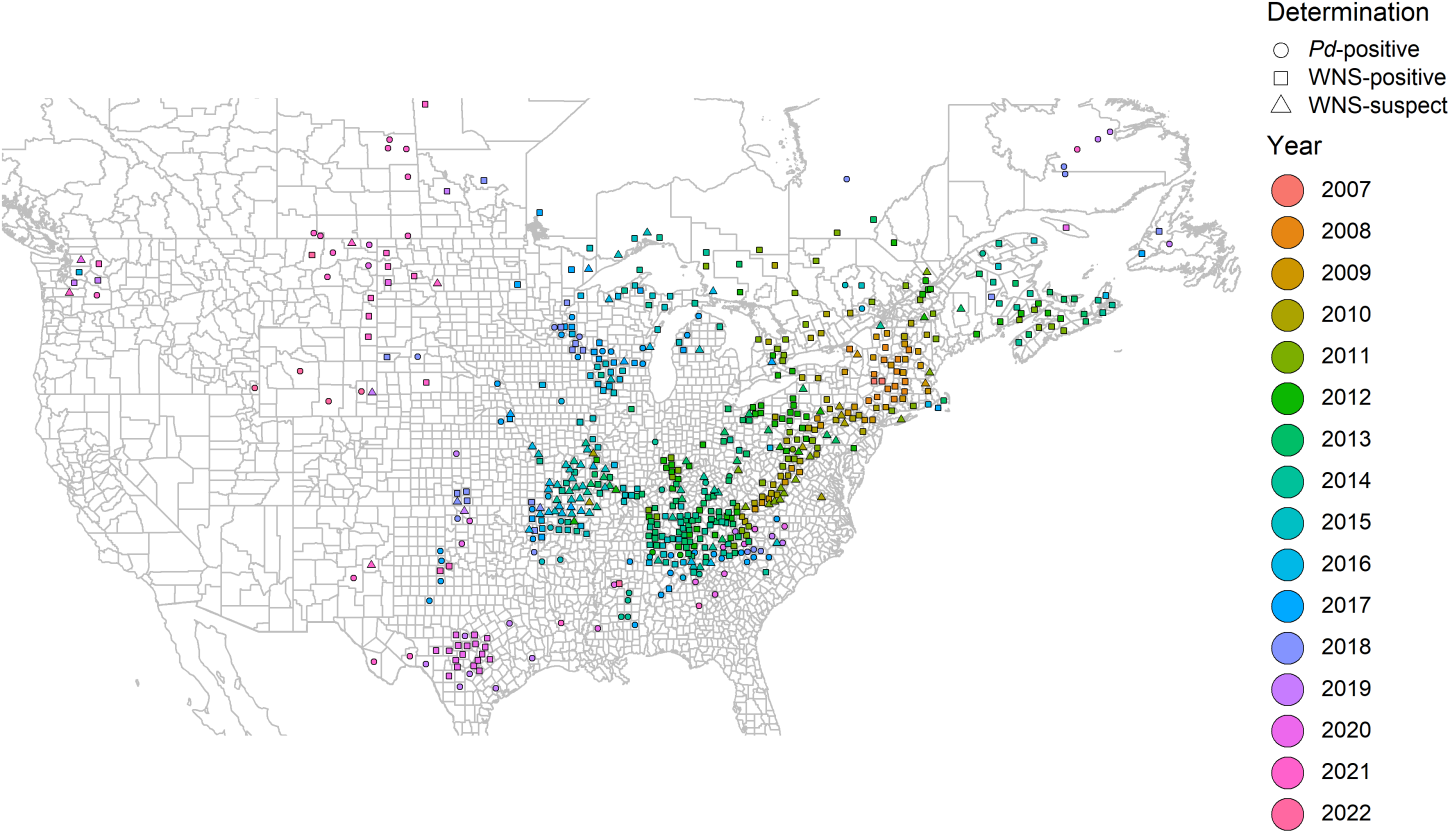
The observation locations, determination status, and the observed year of arrival of WNS or *Pd*. the *Pd* determination status indicates presence of the pathogen whereas WNS determinations were made with a histological examination. Raw data available at https://www.whitenosesyndrome.org.

White‐nose syndrome (WNS) is an emerging infectious disease in North American hibernating bats that has driven widespread population declines and currently threatens several species with local or global extinction (Cheng et al., 2021; Frick et al., 2010; McGuire et al., 2016; Thogmartin et al., 2013; Thogmartin, King, McKann, et al., 2012; White‐nose Syndrome Response Team, 2021a). WNS first emerged in a hibernaculum in upstate New York in 2006 and has since been documented in 48 states/provinces in the United States and Canada (White‐nose Syndrome Response Team, 2020). The pathogen associated with WNS, *Pseudogymnoascus destructans* (*Pd*), continues to spread to new populations and expand its spatial extent each year. Multiple government agencies and research entities have contributed to tracking pathogen invasion and disease emergence at bat hibernacula in the United States and Canada (White‐nose Syndrome Response Team, 2021b). The spatiotemporal nature of these data, now available from the North American Bat Monitoring Program (NABat) database (U.S. Geological Survey, 2021; Loeb et al., 2015) and visualized at https://www.whitenosesyndrome.org (White‐nose Syndrome Response Team, 2020), demonstrates the state of the disease front and its diffusive spread outward from its initial detection (Figure 1).

When WNS arrives at a bat hibernaculum, in most cases the winter survival rate of the population plummets for several consecutive years (Cheng et al., 2021). Based on observed spread over the past decade and a half, it seems likely that the fungal pathogen will continue to spread over the entirety of the United States and possibly the entire continent. Uncertainty remains, however, because although many known sites have been sampled for WNS, many more remain unsampled, in addition to sites still remaining to be discovered. When assessing the status and trend of WNS‐susceptible species, knowing where these populations are in the disease invasion process is crucial (Langwig et al., 2015). For those sites for which abundance monitoring data exist but the presence of WNS or *Pd* has not been determined, these predictions are essential for correctly describing population status. These data can inform models as a covariate in changepoint analyses or for parameterizing vital rates within demographic models (Erickson et al., 2014; Thogmartin, King, Szymanski, & Pruitt, 2012). NABat aims to provide a periodic assessment of species status and trend, and for cave‐hibernating species subject to the deleterious effects of this disease, predictions of disease arrival, with uncertainty, will be crucial for credible assessments. Thus, the primary goal of this work is to produce spatiotemporal predictions of the year of disease arrival within North America, predictions that are readily updatable as new surveillance data become available.

Several studies have attempted to model the spread of *Pd*/WNS across space and time. Various approaches have used statistical models to understand the fundamental processes driving the disease and its effect on animal populations. Inference under these models has yielded estimates of the origin, growth, and spread of the disease, ecological characteristics predicting spatiotemporal disease arrival, and the mortality and behavioral effects it has caused in bat populations. On the continental scale, Thogmartin, King, Szymanski, and Pruitt (2012) use a spatiotemporal, mixed‐effects logistic regression to predict WNS arrival, whereas Maher et al. (2012) and Kramer et al. (2021) modeled the spatial probability of infection using logistic regression gravity models. Meierhofer et al. (2021) investigated the spread of WNS in Texas using host‐pathogen susceptible‐exposed‐infected (SEI) differential equation models.

Wilder et al. (2015) modeled risk of infection among little brown bat *Myotis lucifugus* hibernacula using both geographic proximity and genetic similarity, finding genetic distance improved model fit. Also using genetic data, Thapa et al. (2021) conducted an illuminating phylogeographic analysis on the coat proteins of a partitivirus infecting *Pd* to characterize the spread of WNS across North America. Results trace most infections to a few source locations, including New York, Connecticut, West Virginia, and Kentucky; Thapa et al. (2021) asserted the long‐distance spread to Washington was possibly a human‐mediated transmission from the Mammoth Cave region in Kentucky.

Hefley et al. (2017, 2020) modeled the growth and diffusion of the pathogen using partial differential equations, which provide a convenient method for incorporating science‐based physical models, but numerical algorithms discretizing these equations and performing estimation of parameters can be computationally expensive for even simple spatial models (Hooten et al., 2013; Hooten & Wikle, 2008; Wikle, 2003).

Physical diffusion processes are inherently stochastic. Ecological diffusion models incorporate this physical model, but often include additional elements of uncertainty. For example, the bats most affected by WNS are migratory, but the details about seasonal migrations, the mixing of individuals, and the interaction among species and resulting interspecific spread are not well understood; neither are environmental factors that might mediate spread such as the type of hibernaculum or roosting site (from traditional caves and trees to human‐made structures such as culverts) or the medium of transmission, including the possibility of human‐induced spread. Thus, phenomenological models are useful, particularly when one goal of the analysis is predictive performance. Grider et al. (2021) provides an example prioritizing prediction, using a generalized additive model in the form of a logistic regression on presence of *Pd* including separable, smooth functions of space and time. Results indicate WNS spread is best predicted when including smoothed terms of space and time.

These previous modeling efforts have provided useful information adding to our knowledge of WNS but are not adequate for conservation planning because they are not easily updateable or repeatable. Repeatability can be limited by data availability (e.g. models incorporating logistic regression require presence/absence data and SEI models require host‐pathogen data) or by model complexity (e.g., growth and diffusion models require extensive computational resources and statistical expertise). In particular, Maher et al. (2012) and Meierhofer et al. (2021) mention the importance of cave density and environment. However, this information is not publicly available. In addition, the locations of hibernacula in the western United States are not as well known or documented, limiting the predictive power of such models in the area where predictions are most needed. The uncorrelated Gaussian error structure of these models rely only on covariates to capture the variation in the spread and the anomalous observations such as in Washington, which is often inadequate for predictive purposes.

In the absence of information about cave density and environment or genetic data, a distance‐based model can account for anomalous detections using a generalized error structure. A Gaussian process (GP) can achieve this by including a correlated error structure, modeling local anomalies and subsequent spread, and also possibly by including a residual error structure. The GP is flexible and accurately captures non‐diffusive spread, whereas human‐mediated transmission (e.g., Washington) would not be explained using more covariates such as cave density or environment.

In this study, we use publicly available data reporting year of *Pd* arrival or WNS emergence to develop several GP models that are fast, reproducible, and easily updated with each new iteration of data (U.S. Geological Survey, 2021; White‐nose Syndrome Response Team, 2020). From these models, we obtain a predicted year of *Pd*/WNS arrival over space, as well as inference about the rate of disease spread. Given the rapid spread of *Pd*/WNS and associated effects to bat species, it is critical to provide predictions that can be updated annually at the same pace of the WNS disease cycle and that can be easily reproduced and shared among conservation practitioners.

Although our model is specific to the WNS, the principles underlying our modeling effort are translatable to other disease systems facing similar conservation pressures. Indeed, GPs have been used in epidemiology to capture uncertainties in both the measured data and the underlying disease spread process (Ak et al., 2018; Bhatt et al., 2017; Vanhatalo & Vehtari, 2007). GPs can include ecological and environmental covariates while accounting for additional spatially autocorrelated variation, which can result from either omitted covariates or because ecological processes are often inherently spatial (Wright et al., 2021). Thus, GP models are well‐suited to the *Pd*/WNS monitoring data that are publicly available.

We first present the general model and the variations we implement in Sect 2. We then compare inference and prediction results of the model variations in Sect 3, present a retrospective cross‐validation experiment assessing the predictive accuracy of each model as observations are made over time, and provide details on computational performance. We conclude with a discussion of the significance, limitations, and possible extensions of these results in Sect 4.

## 2 MATERIALS AND METHODS

Each WNS/*Pd*‐monitoring observation in the NABat database includes the year of observed arrival at a given location and the disease determination status (WNS‐positive, WNS‐suspect, *Pd*‐positive, or *Pd*‐presumed), along with geographic coordinates of the site of infection or those imputed to the county level if the site location is considered sensitive information. Most observations are unique to a given county/division, but a handful of administrative regions contain multiple observations at different spatial locations due to their relatively large area. The determination status field encodes both the determination method and result, allowing the aggregation of several data types into a coherent disease designation. WNS results denote the confirmation of disease manifestation, indicating bats were collected and histological examination indicated presence of skin lesions associated with the disease. *Pd* results denote the presence of the pathogen, using swabs taken on bats (mostly live) and tested using the Muller et al. (2013) quantitative polymerase chain reaction (qPCR) protocol to determine fungal load. For our purposes in understanding the spread of this fungal pathogen, we consider only WNS‐positive, WNS‐suspect, and *Pd*‐positive data in this analysis, discarding 13 *Pd*‐presumed observations (compared with 108 WNS‐suspect, 111 *Pd*‐positive, and 398 WNS‐positive) because these are too few observations to inform a model component.

Formally, each observation in the dataset we analyze is indexed by a spatial location s, and the response variable to be modeled is the year of observed arrival of WNS or *Pd* at that location $y\left(s\right)$. The data also include two covariates: let $x\left(s\right)$ be the great circle distance between s and Howes Cave in New York (the location of first detection in North America), and let $w\left(s\right)$ be a categorical variable representing the determination status and take on values *Pd*‐positive, WNS‐positive, and WNS‐suspect (denoting these levels *ω*_1_, *ω*_2_, and *ω*_3_). The data set has *M* = 625 observations sampled between 2007 and 2022.

The four statistical models we compare in this work can be written as variations of the following process model:

$$y\left(s\right)=\mu +x\left(s\right)\beta +z\left(s\right)+\varepsilon \left(s\right)$$

where $\varepsilon \left(s\right)∼\mathrm{GP}\left(0,K_{\varepsilon }\left(s,s^{\prime }\right)\right)$ and $z\left(s\right)∼\mathrm{GP}\left(0,K_{z}\left(s,s^{\prime }\right)\right)$ are mean zero GPs with covariance functions *K*_*ε*_ and *K*_*z*_. A review of GP estimation and prediction can be found in Gelfand et al. (2010), Cressie (1993), and Rasmussen and Williams (2005).

The first of four models that serves as a baseline comparison is the linear model, which is obtained by setting *z* = 0 and white noise covariance $K_{\varepsilon }\left(s,s^{\prime }\right)=\tau^{2}\cdot I\left(s=s^{\prime }\right)$, where I is the indicator function. Using vector notation we can write the data distribution of observations *i* = 1, … , *M* as $y∼ℳ\mathrm{VN}\left(\mu +\mathrm{X\beta},\Sigma_{\varepsilon }\right)$, where μ is a constant length *M* vector with all entries equal to *μ*, *X* is a 1 × *M* vector with *i*th entry equal to $x\left(s_{i}\right)$, and Σ_*ε*_ is the diagonal *M* × *M* matrix defined by the covariance function *K*_*ε*_ with (*i*, *j*)th entry $\Sigma_{\varepsilon }\left(i,j\right)=K_{\varepsilon }\left(s_{i},s_{j}\right)$. The parameters to be estimated are *β* and the residual variance *τ* ^2^.

The mean of the linear model is $E\left[y\left(s\right)\right]=\mu +x\left(s\right)\beta$. *μ* is included as an offset parameter, fixed at the year 2007 for all observations, which implies that *β* represents an unknown spread rate parameter to be estimated in units of years per kilometer (km). In the results section, we report the inverse 1∕*β* in the more familiar units of km/year. This combination of fixed effects was chosen to preserve the interpretation of the effect of distance from Howes Cave as a constant radial spread rate since the introduction of the disease in North America. The model mean is the same for the three GP models about to be described; complexity is added through the second‐order structure of the processes.

The second model, the stationary GP, does not include *ε* but instead includes *z*, a spatially autocorrelated, stationary, mean zero GP with the Matérn covariance function $K_{z}\left(s,s^{\prime }\right)=\sigma^{2}\cdot \frac{2^{1-\nu }}{\Gamma \left(\nu \right)}{\left(\frac{\sqrt{2\nu }d}{\theta }\right)}^{\nu }K_{\nu }\left(\frac{\sqrt{2\nu }d}{\theta }\right)$, where *d* is the great circle distance between two spatial locations of interest s and $s^{\prime }$. The data distribution is $y∼ℳ\mathrm{VN}\left(\mu +\mathrm{X\beta},\Sigma_{z}\right)$, where Σ_*z*_ is the *M* × *M* matrix with (*i*,*j*)^*th*^ entry $\Sigma_{z}\left(i,j\right)=K_{z}\left(s_{i},s_{j}\right)$. The unknown parameters are the correlation range *θ*, the smoothness *ν*, and the autocorrelation variance *σ* ^2^, in addition to *β*.

The third model builds on the stationary GP by adding back in the error process *ε* of the linear model. In geostatistical literature this is often called the nugget effect, so we call this model the nugget GP. The data distribution is

(1) $$y∼ℳ\mathrm{VN}\left(\mu +X\beta ,\Sigma_{z}+\Sigma_{\varepsilon }\right).$$

While spatial variation in the year of arrival due to isolated events can be accounted for using the residual error process *ε*, including a spatially autocorrelated model component *z* allows identification of anomalous regions where the year of arrival was observed either sooner or later than expected based on the constant radial spread rate. Including *z* separates out the influence of these anomalous regions from inference on the spread rate of the disease, the main effect of interest, regardless of whether *ε* is included. Yet including both *z* and *ε* in the third model provides two sources of variation, one correlated and one uncorrelated, about the mean μ+Xβ.

Because disease designation status combines data types conflating disease and fungal sign, variation in spread by designation status may be expected, especially if disease is delayed with respect to fungus arrival. We use disease designation status $w\left(s\right)$ to define the fourth model: the heteroskedastic GP generalizes the white noise error process *ε* in the nugget GP, including heteroskedasticity parameterized using the categorical covariate $w\left(s\right)$. In particular, we define the covariance function

$$K_{\varepsilon }\left(s,s^{\prime }\right)=I\left(s=s^{\prime }\right)\cdot \left[\tau_{1}^{2}\cdot I\left(w\left(s\right)=\omega_{1}\right)+\tau_{2}^{2}\cdot I\left(w\left(s\right)=\omega_{2}\right)+\tau_{3}^{2}\cdot I\left(w\left(s\right)=\omega_{3}\right)\right].$$

The distribution of the data is still given by equation 1, where in this case $\Sigma_{\varepsilon }\left(i,j\right)=K_{\varepsilon }\left(s_{i},s_{j}\right)$ can be defined in practice using the skedastic function $\Sigma_{\varepsilon }=\mathrm{diag}\left(W\tau^{2}\right)$, where $\mathrm{diag}\left(a\right)$ indicates a diagonal matrix with diagonal elements given by vector a, *W* is a *M* ×  3 matrix dummy coding the categorical variable $w\left(s\right)$, and $\tau^{2}={\left(\tau_{1}^{2},\tau_{2}^{2},\tau_{3}^{2}\right)}^{T}$ corresponding to *Pd*‐positive, WNS‐positive, and WNS‐suspect, respectively. This arrangement combines a stationary GP with Matérn covariance with a heteroskedastic residual error process. Model fitting now includes the estimation of $\tau_{1}^{2},\tau_{2}^{2}$, and $\tau_{3}^{2}$ instead of the single variance *τ* ^2^ in the nugget GP. Allowing flexibility among determination statuses via different variance parameters may indicate whether samples taken in proximity but with different determination statuses (or sampling methods) are likely to agree.

In all models, temporal dynamics are modeled in the process mean by the linear predictor defined with *μ* and the radial spread rate *β*, and the remaining variance is partitioned into mean zero processes *ε* and *z* endowed with specific second order structure. Deviations from this radial spread are captured by the spatially correlated GP *z* and the residual error process *ε*. Including a residual error process implies the belief that there is either uncaptured microscale variation due to missing covariates or else observation error in the determination of the year of arrival of *Pd*/WNS. However, this error may not be attributed to the actual observation process in the year of the given observation; for example, consider the possibility that *Pd*/WNS could have arrived in the year(s) prior to the determination and been unobserved during that time. By incorporating residual error components, the surface predictions smooth instead of interpolate the data.

Likelihood methods yield estimates of the fixed effect *β* and covariance parameters, and predictions with uncertainty bounds can be obtained at arbitrary spatial locations for all models. All parameters were estimated except the smoothness parameter in the three GP models, which was fixed at *ν* = 0.5. In R (4.1.0), we use lm to fit the linear model and the optim function to minimize the negative log restricted likelihood function of the stationary, nugget, and heteroskedastic GPs (R Core Team, 2021).

The primary objective of this analysis is to provide the most accurate forecast for the arrival of *Pd*/WNS in terms of predictions and the associated uncertainties, and in this capacity to evaluate the quality of several models and consider model weighting based on metrics of predictive performance. In addition to producing the most up‐to‐date model predictions using the monitoring data for all years (see Figure 1), we assessed the predictive performance of the four models through retrospective cross‐validation. For each year from 2012–2021, we held out that year and all subsequent *Pd*/WNS observations, fit the GP model using only data collected previous to the year in question, and then compared model predictions (with standard errors) to the held‐out data y. Predictions $\hat{y}$ and standard errors $\sigma_{\hat{y}}^{2}$ were obtained using the best linear unbiased predictor for spatial data (Cressie, 1993), the method otherwise known as regression‐kriging or kriging with external drift.

We quantified predictive performance in terms of normalized root‐mean‐square error and the continuous rank probability score, given by $\text{NRMSE}={\left(y-\hat{y}\right)}^{T}\left(y-\hat{y}\right)/\left(\mathrm{IQR}\left(y-\mu \right)\cdot M\right)$ and $\text{CRPS}=\sigma_{\hat{y}}^{2}\left\{\frac{y-\hat{y}}{\sigma_{\hat{y}}^{2}}\left[2\Phi \left(\frac{y-\hat{y}}{\sigma_{\hat{y}}^{2}}\right)-1\right]+2\phi \left(\frac{y-\hat{y}}{\sigma_{\hat{y}}^{2}}\right)-\frac{1}{\sqrt{\pi }}\right\}$, where *M* is the sample size, Φ and *ϕ* are the standard normal cumulative distribution and probability density functions, respectively, and this form is valid for Gaussian predictive distributions only. RMSE is normalized by the interquartile range (IQR) of the response variable excluding the offset term. As more observations are incorporated over time, the range of years of observations and predictions grows larger. Because predictions are made over a larger range, it is expected that RMSE will increase as well. Normalizing by IQR gives a fair comparison of errors across years. CRPS is frequently used when comparing probabilistic forecasts (Gneiting & Raftery, 2007), so we base model weights on the relative values of the reciprocal CRPS.

Prediction intervals may be required if model results are intended to be used in other analyses, possibly as a covariate. Computing prediction standard errors is theoretically straightforward, but storing in memory a large covariance matrix of all locations on a fine spatial grid is prohibitive. Computationally efficient GP models provide an alternative if computing standard errors on the fly is a critical task in the analysis; we do not discuss these models here but see Heaton et al. (2019). In our case, we approximate standard errors by employing the approach of using a subset of one hundred of the nearest neighbors of each prediction location in the computation. This approach circumvents the memory problem and instead we are left with one independent task to perform for each prediction location. We parallelize this computation using the parallel package in R (R Core Team, 2021).

## 3 RESULTS

### 3.1 Status and forecast of WNS

In this section, we present inference and prediction results using all available data between 2007 and 2022 for the stationary GP model. GP models allow us to infer the spread rate of the disease front, identify areas of anomalous time of arrival, and provide isopleth maps with the most up‐to‐date predictions for the spread of *Pd*/WNS. Following in Sect 3.2 are the cross‐validation results which indicate the stationary GP is the best‐performing predictive model. The GP models indicate the disease could cover the coterminous United States by 2030.

The parameter estimates obtained for the linear model and three GP models using all data available are given in Table 1. The fixed effect estimates and confidence intervals in the second column are transformed to the inverse scale in units of kilometers of spread per year to aid in interpretation. Estimates for parameters defining the second‐order structure of each process are given in columns three through eight.

**TABLE 1:** Parameter estimates for 2022 models

Model	1∕*β* (95% CI)	*τ*	*τ*_1_	*τ*_2_	*τ*_3_	*σ*	*θ*
Linear model	182 (178, 187)	2.33	–	–	–	–	–
Stationary GP	182 (173, 191)	–	–	–	–	2.57	79
Nugget GP	207 (164, 282)	1.13	–	–	–	3.34	1239
Heteroskedastic GP	207 (170, 266)	–	2.41	0.61	1.19	2.98	916

*Note*: Units are kilometers per year for 1∕*β*; years for *σ*, *τ*, *τ*_1_, *τ*_2_, *τ*_3_; and kilometers for the correlation range *θ*. *τ*_1_, *τ*_2_, and *τ*_3_ correspond to *Pd*‐positive, WNS‐positive, and WNS‐suspect observations, respectively.

The four models we examined provided increasingly complex insight into disease and fungal spread (Table 1). The linear model indicated the rate of spread was fairly precise at about 182 km/year. The estimate from the GP model without nugget effect was nearly the same but less precise. Conversely, the nugget and heteroskedastic GP models indicated a much more rapid rate of spread (207 km/yr), but prediction intervals were much wider.

The models differed in their estimation of parameters defining the residual error process *ε*. The linear model suggested disease and fungal arrival may have been more than two years earlier or later than observed based on the estimate of *τ*. The residual error estimate for the nugget GP model was smaller, which might be expected as some variance in arrival year in the monitoring observations can be partitioned to the autocorrelated process standard deviation *σ*. The nugget GP estimate of *τ* indicated slightly more than one year residual error, while the heteroskedastic GP model estimates of *τ*_1_,*τ*_2_, and *τ*_3_ indicated arrival may have occurred 2.41 years earlier or later than observed at *Pd*‐positive sites, 0.61 year at WNS‐positive sites, and 1.19 years at WNS‐suspect sites (see Table 1).

Estimates for the spatial autocorrelation parameters *σ* and *θ* have a clear dichotomy between the stationary GP model and the nugget and heteroskedastic GP models. Estimates for *σ* are 15%–30% larger for the nugget and heteroskedastic GPs compared to the stationary GP. The estimated correlation ranges showed an even greater contrast: the estimate for the GP without nugget was 79 km whereas the estimates for the nugget and heteroskedastic GP models exceeded 900 km.

**FIGURE 2:**
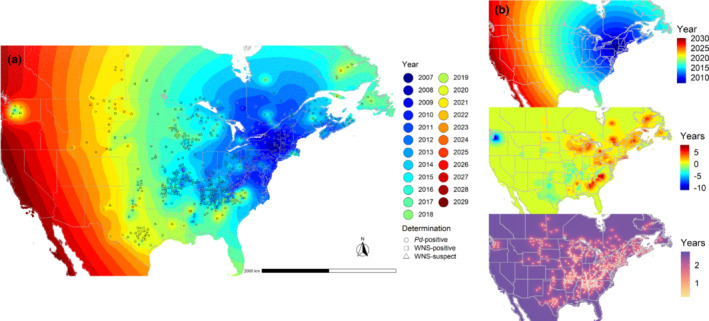
(a) *Pd*/WNS status determination observations and the year of arrival surface predictions on a fine grid for the stationary Gaussian process model using all data from 2007–2022. (b) the top panel shows surface predictions for the stationary GP model including only the offset and radial spread rate fixed effect μ+Xβ. The middle panel shows the spatially autocorrelated variation surface predictions. The bottom panel shows prediction standard errors, excluding residual error, with observation locations indicated by white dots.

For the stationary GP model, we calculated mean predictions and standard errors on a finely spaced grid for visualization (Figure 2). The latent process, excluding the residual error variation, represents the estimated year of arrival of *Pd*/WNS at a given spatial location. See the following section for justification of the choice to display the stationary GP model results. Also see in the supplementary material Figure S3 for predictions from all four models as well as Figure S4 for model‐weighted predictions. Note that plots of surface predictions are rounded to the nearest year because observations were processed in this way, but predictions are actually made on a continuous scale.

Model predictions illustrate the offset and radial spread rate fixed effect in Figure 2b top panel. Surface predictions based on the fixed effects only represent a concentric spread. The basic linear model is clearly deficient in its ability to properly describe deviations observed in the dataset, such as the early arrival in Washington, and later arrivals in the Southeast and Michigan. These deviations are effectively modeled using spatial autocorrelation (Figure 2b middle panel) either with or without the addition of a residual error process.

Based on the surface predictions in Figure 2a, *Pd*/WNS has the potential to cover the entire continental United States by 2030 plus or minus about 3 years based on the prediction standard errors in Figure 2b bottom panel.

### 3.2 Cross‐validation

To assess the retrospective cross‐validation of our models, for each year from 2012–2021 we train the models on all data up to the given year and then perform pseudo out‐of‐sample forecasting on all the data collected the year following the given year.

**FIGURE 3:**
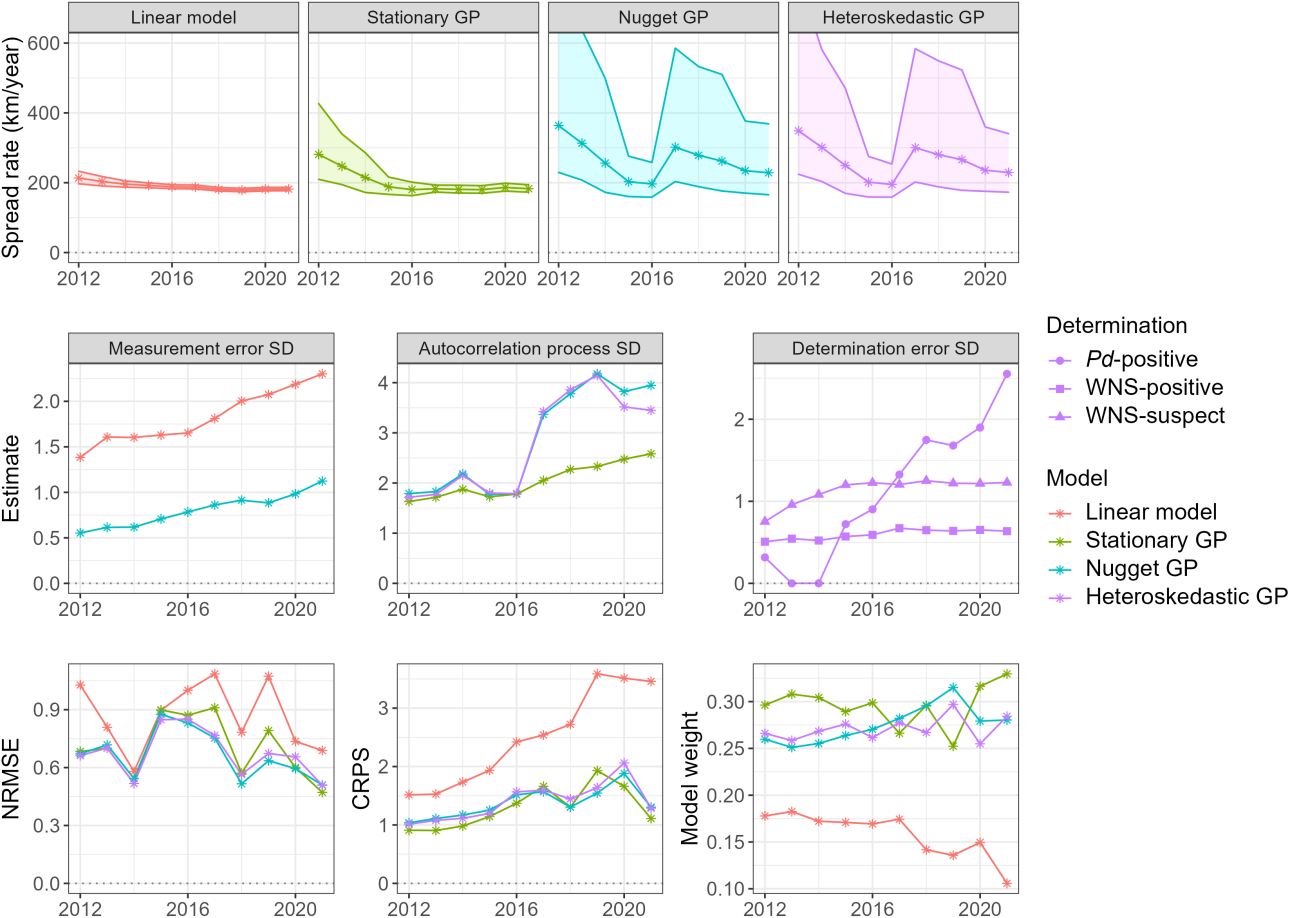
Parameter estimates and performance metrics for annually updated models. Top row shows 1∕*β* for four models. Middle row shows residual error standard deviation *τ*, autocorrelated error standard deviation (SD) *σ*, and heteroskedastic residual error standard deviation components *τ*_1_,*τ*_2_,*τ*_3_, in the units of years. Bottom row shows performance metrics. *τ*_1_, *τ*_2_, and *τ*_3_ correspond to *Pd*‐positive, WNS‐positive, and WNS‐suspect observations, respectively.

Estimates of the spread rate for the four models converged on 150–350 km/year (with uncertainty bounds) for all four models by 2021 (top row of panels in Figure 3). The middle row of Figure 3 shows standard deviation parameter estimates over time (in units of years), with *τ* in the first column, *σ* in the second column, and *τ*_1_, *τ*_2_, and *τ*_3_ in the third column. The bottom row of Figure 3 shows the NRMSE, CRPS, and model weights of each model over time.

The largest residual error standard deviation (*τ*'s) was estimated in the linear model, expectedly, because there is no spatial autocorrelation model to help absorb excess deviations from the fixed effect. Both the linear and nugget GP models display consistent, slightly increasing error standard deviation estimates over time because more observations are used to fit the model.

The heteroskedastic standard deviation estimates (*τ*_1_,*τ*_2_,*τ*_3_) give some insight into how the different determination status data types are integrated into model predictions. *Pd*‐positive data were first collected in 2011 and makes up an increasing fraction of data collected over time (see Figure S2). The bottom right panel of Figure 3 shows the *Pd*‐positive determination status standard deviation *τ*_1_ increases over time as more observations are collected and becomes larger than *τ*_2_ and *τ*_3_. These results align with the fact that *Pd*‐positive results (absent WNS confirmation) allow us to infer the presence of the fungus before the disease. Therefore, we would expect *Pd*‐positive observations to deviate consistently from the disease front with standard deviation *τ*_1_ (in years). None of the residual error standard deviations (*τ*'s) decrease over time for any model, indicating the monitoring data continue to deviate from model predictions, with positive cases found outside of the predicted disease front. These observations show the value of using a determination status skedastic function within the GP model. However, approximate standard errors about the parameter estimates from the observed Fisher information matrix are large and more data are needed to make any definite conclusions on whether there are statistically significant differences among the heteroskedastic variance components.

The spread rate 1∕*β* was fairly consistent and became more precise over time for the linear model. For the stationary GP model the spread rate estimates were larger for early years, then converged on the linear model estimates but with less precision. The nugget and heteroskedastic GP models exhibited a decrease in spread rate estimates until 2016 at which point it showed a marked increase followed by a subsequent decrease thereafter (top row of panels, Figure 3). This spike could be due to the marked increase in *Pd*‐positive data informing the model following 2016 (see below). With one exception, all the sources of model error generally increased over time as the disease spread farther across the continent (bottom row of panels, Figure 3); WNS‐positive sites were fairly invariant in their level of uncertainty over time, whereas WNS‐suspect sites increased in error early (before 2016) before leveling off. In 2017, *Pd*‐positive site error increased greatly, coinciding with a 2.5‐fold increase in *Pd*‐positive determinations made (Figure S2).

Including model components in addition to the uncorrelated error in the linear model decreases prediction NRMSE and CRPS, with the more flexible models capable of better predictive accuracy and precision. Predictive performance metrics indicated the linear model performed less well than the three GP models (middle row of panels, Figure 3). Model weights by 2017 indicated the GP models each have about twice the weight of the linear model; there is little evidence for preferring one GP model over the other, however, as they perform similarly over time.

Including both *z* and *ε* (in the nugget and heteroskedastic models) allows the variation about the mean to be partitioned into two separate sources. Therefore, one might expect estimates of *σ* ^2^, the variance of autocorrelation process *z*, to be smaller in the nugget and heteroskedastic GP models compared to the stationary GP model. In addition, the increased flexibility can improve predictive performance. Unexpectedly, estimates of the autocorrelated variation magnitude and length scale (*σ* and *θ*) are larger when *ε* is included in the nugget and heteroskedastic GP models than in the stationary GP model. By including a residual error component that can account for differences between the observations and model predictions, the estimated spatial process is required only to account for a part of the error between observations and model predictions. In this case, the addition of the residual error component may relieve the spatial process from accounting for all of the prediction error and give more accurate estimates of the parameters. Despite this explanation, based on the cross‐validation experiment and the predictive metrics we examined, it seems the addition of *ε* in the nugget and heteroskedastic GP models slightly worsens predictive performance. Because it has performed the best over the past 3 years in cross‐validation, the stationary GP model results are given in Sect. 3.1.

This cross‐validation experiment reveals how model estimates evolve over time as new data are collected. Model weights calculated from the CRPS predictive performance metric indicated which models might be useful for making predictions. Model averaging using these weights provides one way to combine models with several hypotheses into probabilistic predictions (Nichols et al., 2021). Figure S3 shows predictions using all available data from 2007–2022 for the four models we investigated, and Figure S4 combines these predictions weighted by the 2021 cross‐validation weights.

All stationary GP models took under a minute to fit, and all heteroskedastic GP models took less than 2.5 minutes to fit on a Dell Precision 7550 with 10th Generation Intel Core i9‐10885H vPro processor. Predictions on a fine spatial grid with over 180,000 spatial locations took about 1.5 minutes for all models, and prediction intervals on the same grid took under 45 minutes using 16 cores for all GP models fitted using monitoring data from all years.

## 4 DISCUSSION

In this study, we investigated several Gaussian process models providing spatial smoothing of publicly available *Pd*/WNS monitoring data, supplying a temporal forecast of disease spread on a continental spatial scale. The GP models we developed in this study present several advantages to conservation practitioners: they provide a fast and easily implemented model for the spread of *Pd*/WNS, are capable of prediction and uncertainty estimates at arbitrary locations, and retain the interpretability of model components, which is useful for visualization and communication of results to a nontechnical audience or the general public.

Model fitting and prediction is computationally tractable when combining GPs with maximum likelihood estimation and a modest‐sized dataset (625 observations). Model fitting and prediction can be achieved within a few minutes, and these timing results are presumably comparable to using regression‐based models of Maher et al. (2012), Thogmartin, King, Szymanski, and Pruitt (2012), Kramer et al. (2021), and Grider et al. (2021), while outperforming more complex mechanistic models of Hefley et al. (2017, 2020).

The models we developed are capable of predictions of the time of arrival of *Pd*/WNS at locations where the disease has not yet been observed. More generally, model predictions on a fine spatial grid can provide isopleth maps showing disease progression at annual increments, forecasting the spread of *Pd*/WNS throughout North America. Programmatically, this work provides capacity for NABat (Loeb et al., 2015) with predictions incorporated into annual status and trend assessments of bat species; as new observations become available, the speed and ease of calculation allow rapid incorporation into assessments. Quantitative results from models are a key component of structured decision‐making and adaptive resource management (Nichols et al., 2021; Runge et al., 2020). Being able to predict disease arrival could also help inform preventative measures to protect bat populations (Grider et al., 2021; Meierhofer et al., 2021). These results benefit policymakers, researchers, and the public by providing a better understanding of what the future outlook for bats may look like, and what conservation actions may need to be taken to protect these species (Bernard et al., 2020).

In particular, many studies have attempted to develop and model potential WNS intervention strategies (Duan et al., 2021; Fletcher et al., 2020; Palmer et al., 2018; Rocke et al., 2019; Turner et al., 2022). These approaches, however, can be hampered by a paucity of information on timing of disease occurrence. Potential management and implementation of these strategies can be more effective if applied before or along the disease front. Knowing where the disease is currently and where it is likely to be in the near future can be informed by our model predictions.

The spread of wildlife diseases are complicated phenomena regulated by the available network of transmission and other ecological factors. Compared to previous statistical approaches that model disease occurrence as a function of space and time, the response variable of interest here is the year of arrival, indexed only in space. The combination of a temporal response and distance covariate implies the estimated fixed effect is the rate of disease spread, which is a meaningful measure for practitioners. The simple structure of the GP models we utilized ensured we did not have to sacrifice interpretability of model parameters, whereas in logistic regressions fixed effect estimates and rates can be cumbersome to communicate in units of probabilities of occurrence. These estimates are then often subsequently thresholded to produce forecasts similar to isopleth maps. The second‐order model components of the GP models can also be interpreted within the epidemiological context. The residual error, for example, could represent measurement error or microscale variation from missing covariates, and regions with spatially correlated deviations from the estimated spread rate are attributable to anomalous early or late disease arrival. Another advantage of this simple approach is that the only pieces of information needed to predict to unobserved locations are the spatial coordinates and the distance from the disease epicenter, which is easily calculated and time invariant. Our exclusion of additional covariates at this time is intentional for two reasons: predictions requiring these covariates may limit their spatial or temporal support, and the meaning of spread rate within the model can become obscured, especially with the inclusion of nonlinear functions of space and time.

However, the autocorrelation process *z* in the GP models is, however, able to account for deviations from a constant spread rate without additional covariates. The slow spread estimates of the GP model seems to be accurately capturing the observed spread exhibited by the data. This is a boon for using a GP model to include a spatial autocorrelation process in comparison to competing regression‐based models based on covariates alone without a generalized error structures. The GP models are able to incorporate multiple components of variation existing within the monitoring data through the autocorrelated and uncorrelated processes *z* and *ε*. The different estimates of the extent of spatial autocorrelation in the data measured by *σ* and *θ* are illuminated by comparing the stationary GP with the nugget and heteroskedastic GPs. In the heteroskedastic model, differences in residual standard deviation estimates indicate how data from each of the three determination statuses contributed to the spatial identification of the disease front.

These results tell a compelling story regarding the spread of *Pd*/WNS in North America, identifying areas significantly deviating from the concentric spread such as the late arrival in the Southeast and early arrival in Washington, where the spread may have been human‐mediated. As the spread of the disease becomes more erratic over time, however, anomalies such as that on the eastern coastal plain may not be completely captured by the model. One possible explanation is that testing improved over time, allowing *Pd* to be detected sooner, but during the initial years of model fitting, the model was built solely on observations of WNS. Only using WNS positive or suspect cases in early sampling likely resulted in artificially low rates of Pd spread. This possibility is reinforced by the decrease in spread rate estimates observed until 2016 before a sudden increase (Figure 3) and the concurrent increase in *Pd*‐positive determinations and increase in *Pd*‐positive site error. These observations are apparent because of and may indicate the usefulness of the heteroskedastic GP model. However, because any one GP model does not vastly outperform the others (except the deficient linear model), model weighting/averaging (Figure S4) may be a useful compromise when making future predictions. Model‐weighted predictions would benefit from the inclusion of models of competing hypotheses and assumptions, such as gravity and ecological diffusion models, and future work may explore how predictions from models with different structures and response variables can be integrated in this framework.

The choice of model used for disease spread ultimately depends on the required output. The GP models we present can be readily fitted on an annual basis as new data become available. However, if fine‐grained spatial information is required about fungal growth or diffusion, an ecological diffusion model may be preferable.

The spatiotemporal prediction forecasts we produced are valuable to those studying bats in areas where *Pd*/WNS has yet to arrive but may have a significant effect in the future, such as the southwestern United States. Several features that distinguish the ecological niche of bats in the western United States make it difficult to make forecasts in this region: there may be fewer WNS‐susceptible species and bats hibernate at lower densities often in unconventional, inaccessible, or unknown sites (Bogan et al., 2003; Hendricks, 2012). In addition, the dynamics of the spread from the long‐distance transmission to Washington are still unfolding and contributes to uncertainty in the disease front in the West. In efforts not shown, we explored one model variation including a distance covariate from the first observation in Washington, but this model variation did not appreciably improve model fit or performance and thus was not explored further; in future years, however, this model remains a viable candidate for further examination.

## AUTHOR CONTRIBUTIONS

**Ashton Wiens:** Conceptualization (equal); formal analysis (lead); investigation (equal); methodology (lead); software (lead); validation (equal); visualization (equal); writing – original draft (lead); writing – review and editing (equal). **Wayne Thogmartin:** Conceptualization (equal); investigation (equal); supervision (lead); validation (equal); writing – review and editing (equal).

## FUNDING INFORMATION

U.S. Geological Survey, Ecological Sciences Branch, Ecosystems Mission Area, Species Management Research Program.

## Supporting information


Figure S1.

Figure S2.

Figure S3.

Figure S4.

## Data Availability

Code and model results are available at https://doi.org/10.5066/P9ZD9GVZ and https://doi.org/10.5066/P9XYRQ1K, respectively.

## APPENDIX A

Model estimates for standard deviation parameters shown in the bottom row of Figure 3 generally increase over time as more observations over a larger spatial domain are used to fit the models. To investigate the effect of the increasing area of the spatial domain on the magnitude of the standard deviation parameters, we plot the ratio of each standard deviation parameter to the area of the spatial convex hull of observations in Figure S1. These area weighted standard deviation estimates generally decrease initially before stabilizing over the final 5 to 10 years. Stable estimates may indicate these errors arise from irreducible background variability emerging from the monitoring data. If this is the case, more observations will increase the predictive performance of this approach but will not diminish the size of the irreducible errors. Stable estimates of standard deviation parameters over time similarly yields stable uncertainty quantification about model predictions, based on the monitoring tools, datasets, and the methods we used to conceptualize and model the problem.

Figure S2 shows the number of determination status observations in the dataset each year. Note the first appearance of *Pseudogymnoascus destructans* (*Pd*) determination data in 2011 and the marked increase in 2017 and the following years.
