# Supplementary figures and images for: Gaussian process forecasts Pseudogymnoascus destructans will cover coterminous United States by 2030
Source: Ecol Evol. 2022 Nov 27;12(11):e9547. doi: 10.1002/ece3.9547 (PMC9702997; doi:10.1002/ece3.9547)

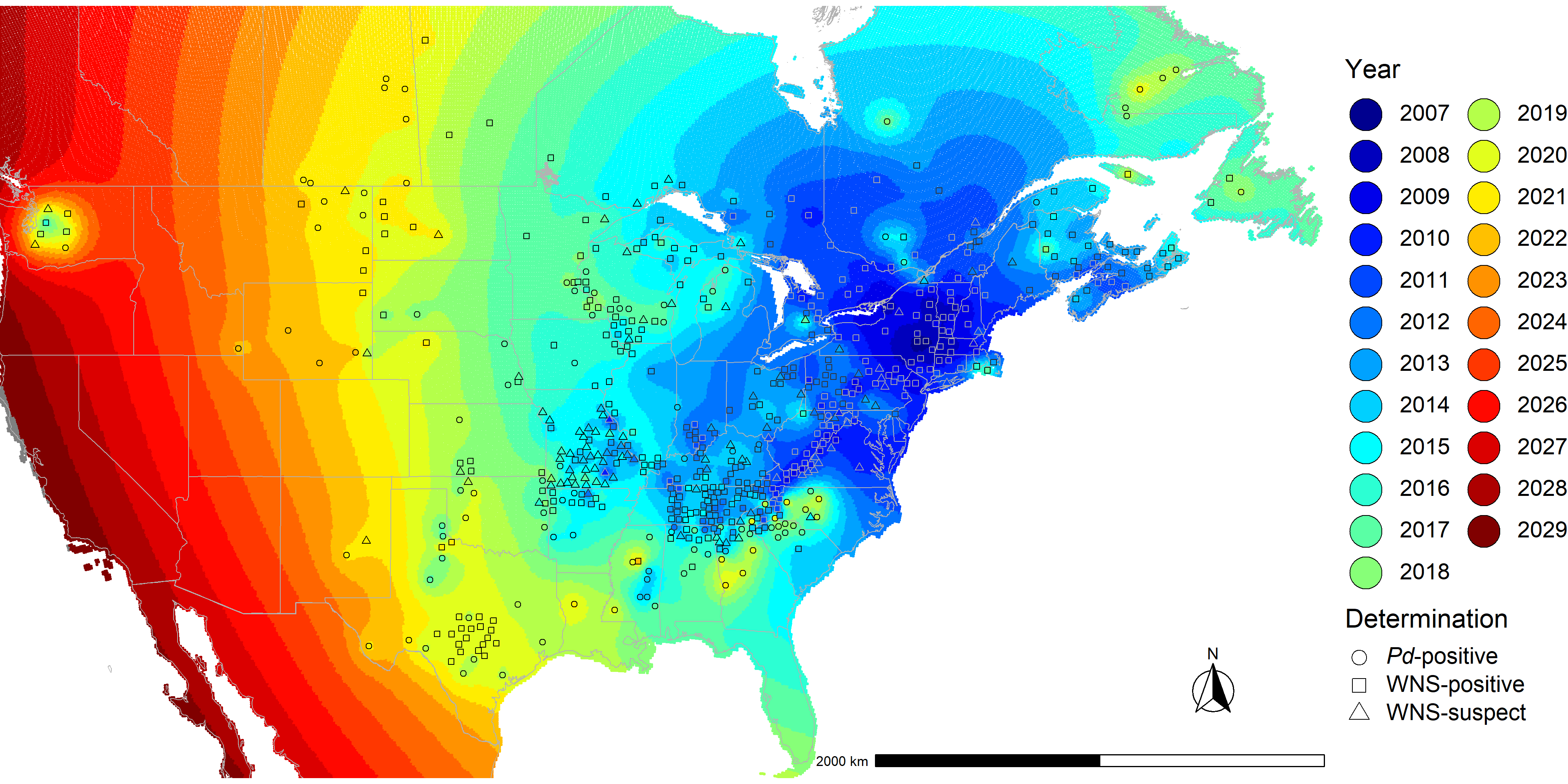

Supplement: Supplementary file 1 — Figure S1. [file ECE3-12-e9547-s005.png]

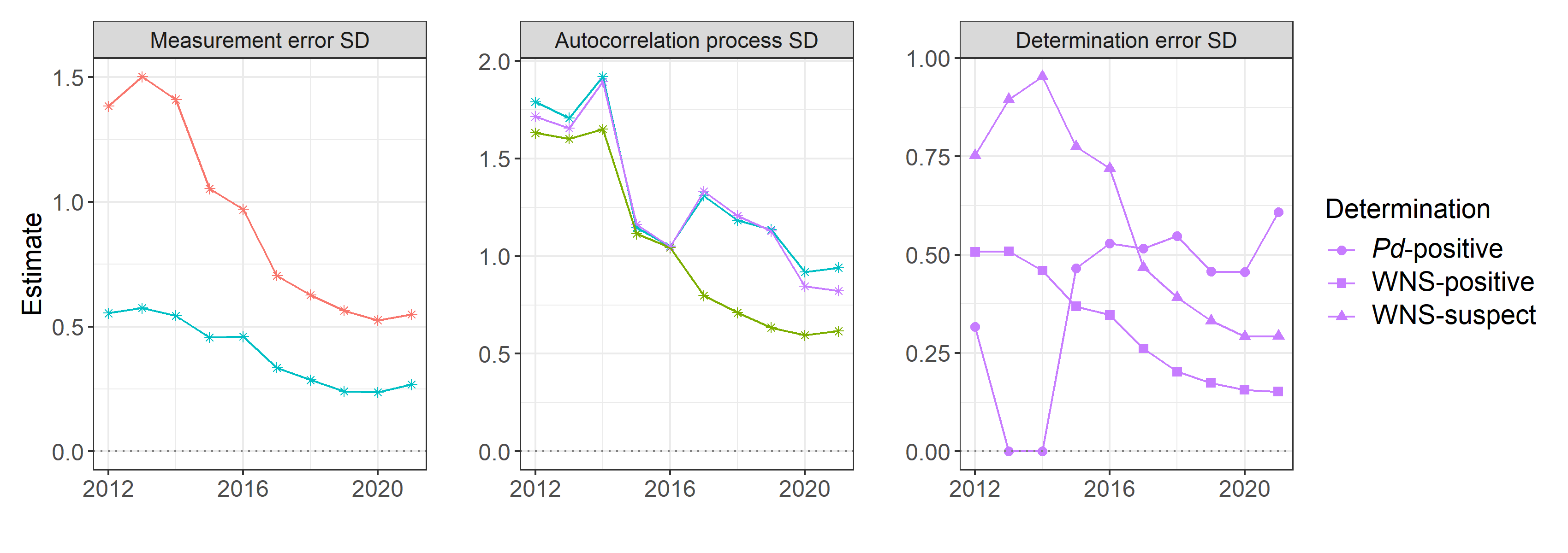

Supplement: Supplementary file 2 — Figure S2. [file ECE3-12-e9547-s002.png]

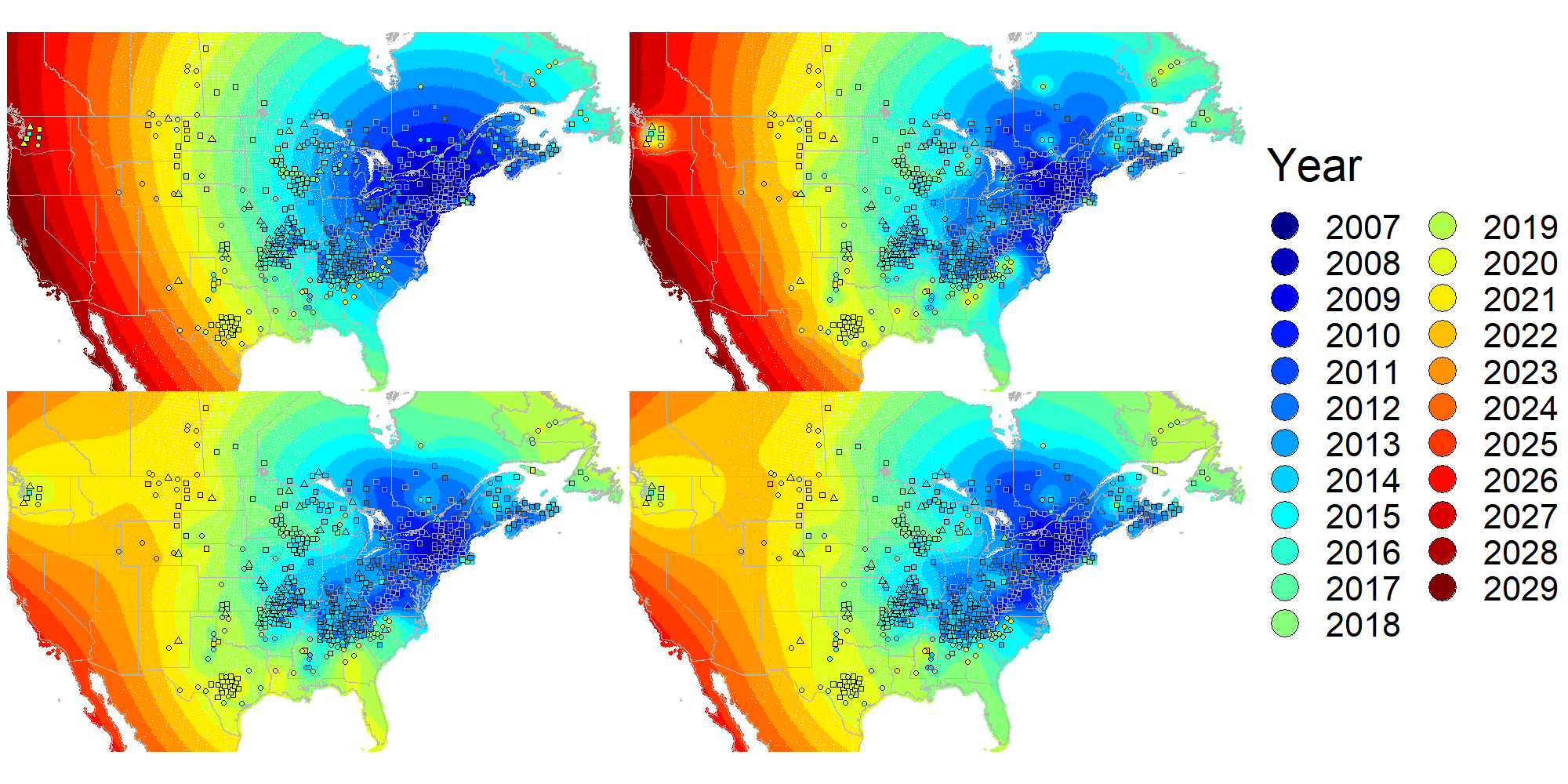

Supplement: Supplementary file 3 — Figure S3. [file ECE3-12-e9547-s003.png]

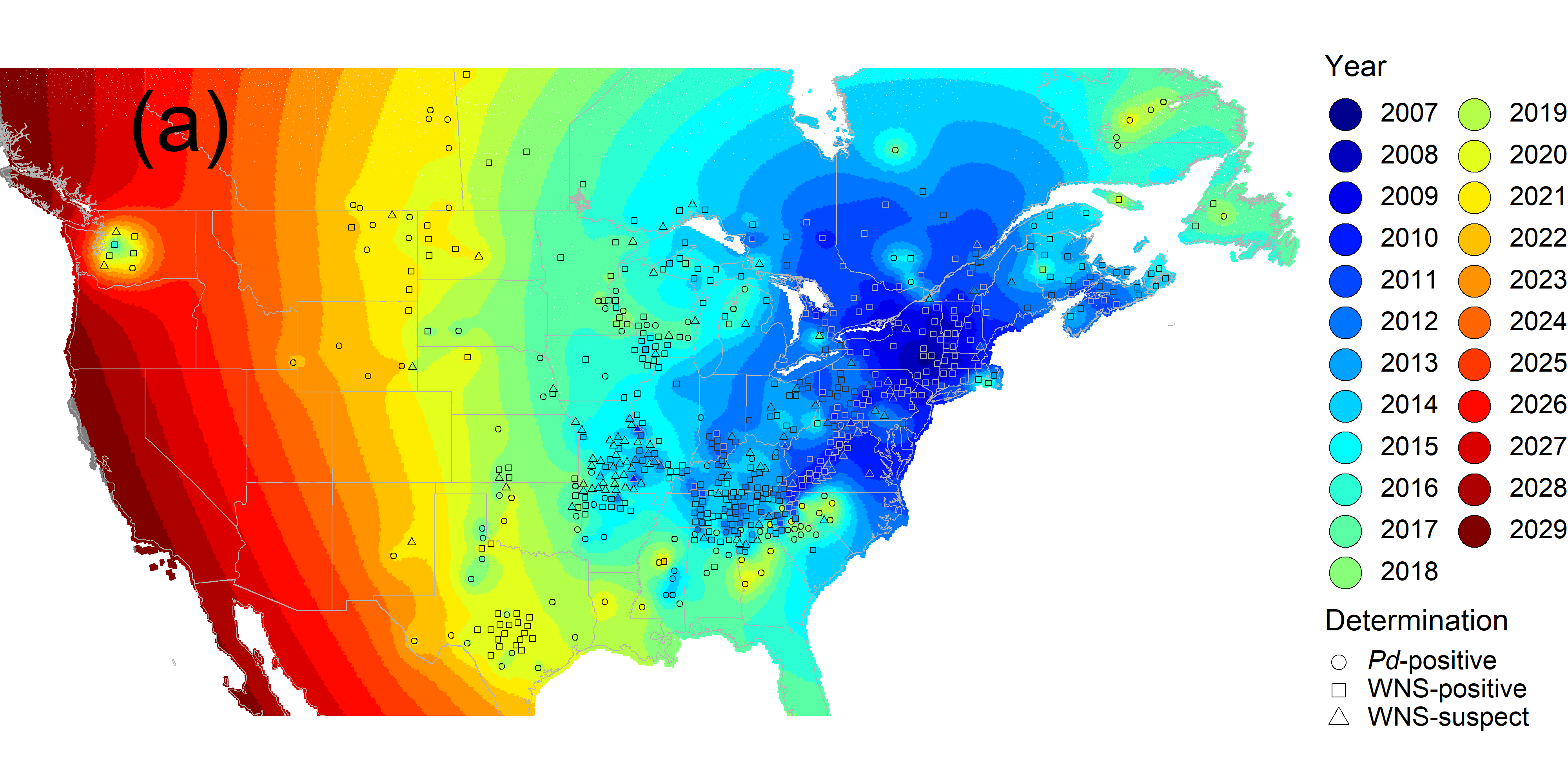

Supplement: Supplementary file 4 — Figure S4. [file ECE3-12-e9547-s004.png]
