# Supplementary material for: Gaussian process forecasts Pseudogymnoascus destructans will cover coterminous United States by 2030
Source: Ecol Evol. 2022 Nov 27;12(11):e9547. doi: 10.1002/ece3.9547 (PMC9702997; doi:10.1002/ece3.9547)
Supplement: Supplementary file 5 [file ECE3-12-e9547-s001.docx]

**Figure S1:**

The number of determinations (observations) collected per year in the dataset for each of the three determination statuses examined in this analysis.

**Figure S2:**

Standard deviation parameter estimates for annually updated models, scaled by the area of the spatial domain (the convex hull of the observation locations). Left panel shows area weighted $\tau$, middle shows area weighted $\sigma$, and right shows area weighted $\tau_1, \tau_2, \tau_3$, which correspond to \textit{Pseudogymnoascus destructans} (\textit{Pd})-positive, white-nose syndrome (WNS)-positive, and WNS-suspect observations, respectively. All areas were in addition divided by the minimum convex hull area $2.02\times10^{12}$ meters$^2$; thus, units for all panels are years/$(2.02\times10^{12})$ meters$^2$.

**Figure S3:**

White-nose syndrome/\textit{Pseudogymnoascus destructans} status determination observations and the modeled year of arrival surface predictions on a fine grid for the linear model (top left), stationary GP (top right), stationary GP with nugget effect (bottom left), and stationary GP with heteroskedastic nugget (bottom right).

**Figure S4:**

Model-weighted year of arrival of white-nose syndrome/\textit{Pseudogymnoascus destructans} surface predictions on a fine grid, combining the predictions in the four panels of Fig. \ref{fig:fourmodelpredictions} with the 2021 model weights (Fig. \ref{fig:estimates}, right panel of middle row)
